# Transcriptome sequencing and annotation of the microalgae *Dunaliella tertiolecta*: Pathway description and gene discovery for production of next-generation biofuels

**DOI:** 10.1186/1471-2164-12-148

**Published:** 2011-03-14

**Authors:** Hamid Rismani-Yazdi, Berat Z Haznedaroglu, Kyle Bibby, Jordan Peccia

**Affiliations:** 1 Department of Chemical and Environmental Engineering, Yale University, New Haven, CT 06511, USA; 2 Department of Chemical Engineering, Massachusetts Institute of Technology, Cambridge, MA 02139, USA

## Abstract

**Background:**

Biodiesel or ethanol derived from lipids or starch produced by microalgae may overcome many of the sustainability challenges previously ascribed to petroleum-based fuels and first generation plant-based biofuels. The paucity of microalgae genome sequences, however, limits gene-based biofuel feedstock optimization studies. Here we describe the sequencing and *de novo *transcriptome assembly for the non-model microalgae species, *Dunaliella tertiolecta*, and identify pathways and genes of importance related to biofuel production.

**Results:**

Next generation DNA pyrosequencing technology applied to *D. tertiolecta *transcripts produced 1,363,336 high quality reads with an average length of 400 bases. Following quality and size trimming, ~ 45% of the high quality reads were assembled into 33,307 isotigs with a 31-fold coverage and 376,482 singletons. Assembled sequences and singletons were subjected to BLAST similarity searches and annotated with Gene Ontology (GO) and Kyoto Encyclopedia of Genes and Genomes (KEGG) orthology (KO) identifiers. These analyses identified the majority of lipid and starch biosynthesis and catabolism pathways in *D. tertiolecta*.

**Conclusions:**

The construction of metabolic pathways involved in the biosynthesis and catabolism of fatty acids, triacylglycrols, and starch in *D. tertiolecta *as well as the assembled transcriptome provide a foundation for the molecular genetics and functional genomics required to direct metabolic engineering efforts that seek to enhance the quantity and character of microalgae-based biofuel feedstock.

## Background

Global demand for petroleum as a transportation and heating fuel is predicted to increase 40% by 2025 [1]. Liquid biofuels from plants and microalgae feedstock represent a renewable sustainable alternative to petroleum energy when greenhouse gases released during the combustion of these biofuels are partially neutralized by the carbon dioxide required for their growth. The greatly minimized acreage estimates, high lipid or starch content, and biomass production rates that surpass those of terrestrial plants suggest that biodiesel or ethanol derived from lipids or starch produced by microalgae may circumvent many of the limitations ascribed to petroleum fuel and first generation plant-based biofuels [2-7]. The most commonly stated paradigm for producing biodiesel from microalgae is to grow these microorganisms in open pond or closed reactor systems, extract the lipids or starch, and transform them into biodiesel by transesterification or ethanol by fermentation, respectively.

Unlike ethanol or other plant biofuels, technology to economically grow microalgae with high lipid or starch content is in the early stages of development [2]. Economic viability and environmental sustainability require the optimization of characteristic microalgae strains and ecologies in order to increase the per cell enrichment of lipids or starch and to improve fuel production and performance properties [6]. An in-depth knowledge of microalgae genomics precludes these necessary increases in biological efficiency. Numerous studies concerning the effects of stress conditions on lipid and starch contents of microalgae have been documented in the literature [8-14]. However, an understanding of how microalgae respond to physiological stress at molecular level is largely limited to model organisms [15,16], and the relevant pathways in microalgae have not been fully documented [17]. Although transcribed gene and pathway information is requisite for planning and introducing stable and successful genetic manipulations in these microalgae, these efforts have been hampered by the lack of sequenced genomes of biofuel relevant microalgae. Due to the large efforts that are required to sequence these medium size (~100 mb) eukaryotic genomes, only seven microalgae genomes have been completed as of 2010 [7]. Alternatively, transcriptome sequencing can be a more efficient approach for obtaining microalgae functional genomics information. Transcriptome sequencing targets only coding DNA and this reduced sequencing requirement coupled with the rapidly evolving next-generation sequencing methods can result in high transcriptome coverage depth and facilitates the *de novo *assembly of transcriptomes from species where full genomes do not exist [18-21]. The more rapid and economic creation of these transcriptomes enables researchers to focus on organisms of direct biofuels interest and reduce the reliance on model organisms [19].

The objectives of this study are to discover genes that encode enzymes involved in the biosynthesis of biofuel precursors in the microalgae *Dunaliella tertiolecta *and to describe the relevant metabolic pathways. *D. tertiolecta *is a flagellated unicellular marine microalga belonging to the Chlorophyta phylum. The rational for selecting *D. tertiolecta *as a non-model organism in this study lies in its ability to produce large quantities of lipids and starch (up to 67% and 27% of organism dry weight, respectively), rapid growth rate in hyper saline environments which aids in maintenance of pure cultures, and lack of a rigid cell wall which eases product extraction and genetic manipulation [22-27]. These unique physiological and structural traits gives *D. tertiolecta *considerable advantages over model organisms such *Chlamydomonas reinhardtii *as feedstock for biofuel production.

*D. tertiolecta *was cultured under nitrogen- and osmotic-inducing stress conditions and total RNA was extracted from cells during log and stationary growth phases. Libraries of cDNA constructed from total RNA were normalized and sequenced using the 454 GS FLX platform with Titanium chemistry. The transcriptome was assembled using the pool of sequencing data obtained from all cDNA libraries, and resulting individual transcripts (isotigs) and singletons were annotated. Sequences were screened to identify enzymes-encoding genes present, and relevant lipid and starch pathways were reconstructed. Results demonstrate the capability of using transcriptome data from next-generation sequencing to identify pathways of interest and potential targets for metabolic engineering in microalgae, and enable functional genomics studies on a non-model species relevant for the production of next-generation biofuel.

## Results and Discussion

### Sequencing and *de novo *assembly of the transcriptome

To identify genes and reconstruct the metabolic pathways involved in the production of biofuel precursors in *D. tertiolecta*, pure cultures were grown under nitrogen rich and nitrogen depleted conditions, and high salt concentrations. Cells were harvested in the log and stationary growth phases. These conditions are known to influence the production and accumulation of lipids and starch in microalgae [23,28-30], and were therefore used to increase the expression and maximize the diversity of genes related to these processes. Responses for nitrogen deprivation resulted in starch concentration doubling to over 25% of the cell dry weight with no increase in lipid content. The elevated salt concentration did not affect the starch content of nitrogen sufficient cells harvested during the stationary phase, but resulted in 22% increase in the total lipid content of the cells. Harvesting of microalgae in the exponential growth phase resulted in a near doubling of the lipid content to greater than 35% of the cell dry weight versus the stationary phase. The normalized cDNA libraries of cells grown under the above conditions were pooled and sequenced using the 454 GS FLX Titanium, and the *D. tertiolecta *transcriptome was assembled from the resulting sequencing reads.

**Figure 1:**
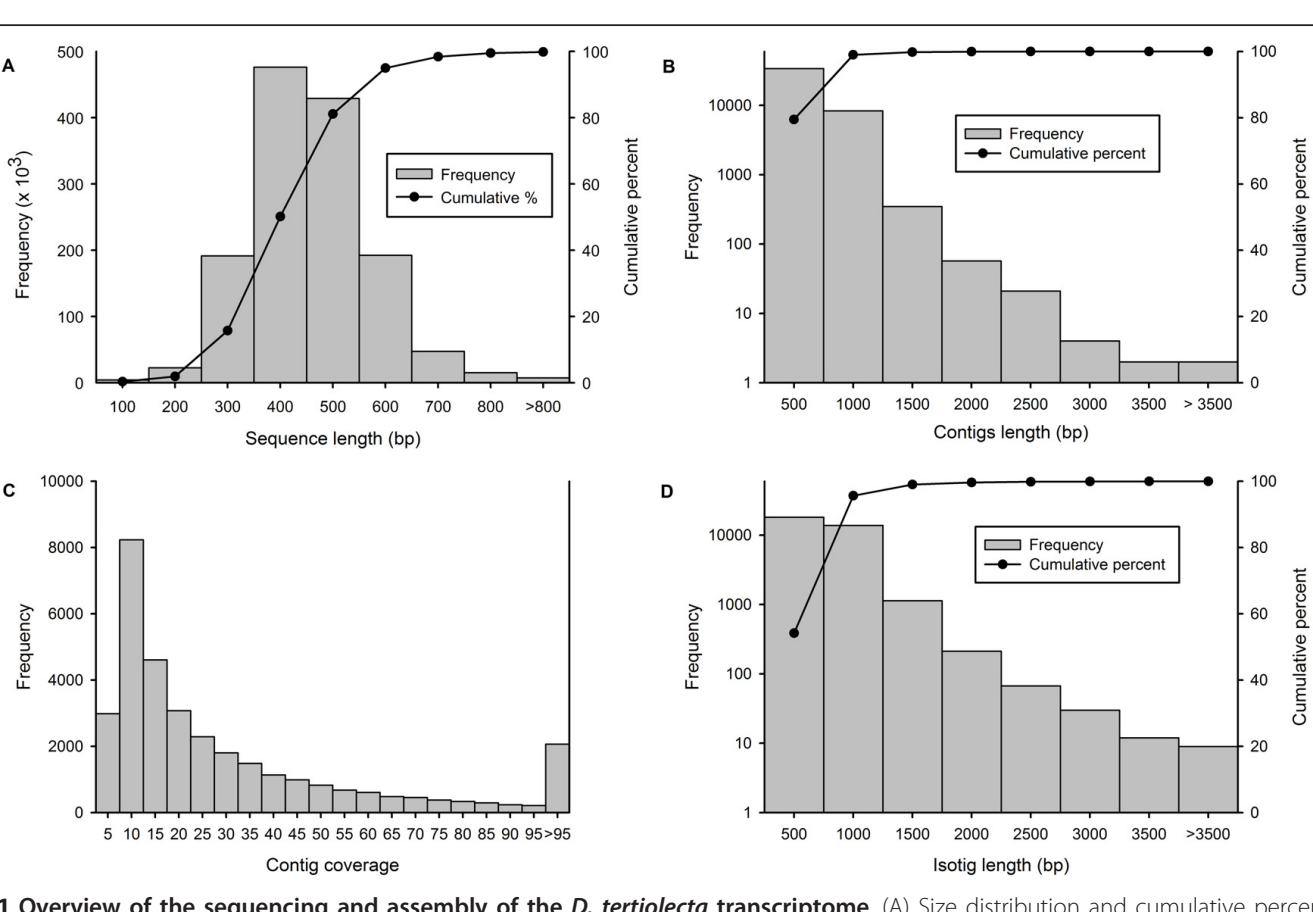
**Overview of the sequencing and assembly of the *D. tertiolecta *transcriptome**. (A) Size distribution and cumulative percentage of raw sequence reads, (B) size distribution and cumulative percentage of contigs, (C) distribution of contigs sequence coverage, (D) size distribution and cumulative percentage of isotigs.

Sequencing of cDNA libraries generated a total 1,385,389 raw reads, with an average length of 410 bp. The size distribution of raw reads is shown in Figure 1A, and a summary of sequencing and assembly results are presented in Table 1 and Additional file 1. After trimming for the adaptors and primer sequences, 20,036 sequences were removed due to their short length, low complexity, and overall low quality scores. This pre-assembly cleaning and trimming step resulted in 1,365,353 high quality (HQ) reads, with an average length of 400 bp, corresponding to 98.5% of the original raw sequences.

**Table 1:** D. tertiolecta transcriptome sequencing and assembly summary

	Sequences	Bases (Mbp)
Sequencing		
Raw sequencing reads	1,385,389	567.6
Average read length	410 bp	
		
Pre-assembly		
Trimmed	352,422	
Trashed	20,036	
Reads used in assembly	1,365,353	546.6
Average read length	400 bp	
		
^1^Assembly		
		
Contigs		
Reads assembled as contigs	609,149	258
Number of contigs	34,301	
Average length of contigs	377 bp	
Range of contigs length	86-4,258 bp	
Depth on contigs	31	
		
Isotigs		
Number of isotigs	33,307	17.8
Average length of isotigs	535 bp	
Range of isotigs length	101-4,941 bp	
Depth on isotigs	2.1	
		
Singletons	376,482	70.1
Unique sequence	409,789	

^1^While this manuscript was in the review process, Newbler v2.5 became publicly available. We reassembled the *D. tertiolecta *transcriptome using Newbler v2.5 and the outcomes of assembly in comparison with those obtain from v2.3 are presented as the Additional file 1. The differences observed between the outputs of two versions were not significant to impact the major or minor conclusions of this study, and are consistent with reports from Ewen-Campen et al. [77].

Trimmed and cleaned sequences were assembled using the cDNA assembly feature of Newbler software v.2.3. (Roche, IN, USA). A total of 609,149 HQ reads were assembled into 34,301 contiguous sequences (contigs), and 376,482 reads were identified as singletons (i.e., reads not assembled into contigs). The size of contigs ranged from 86 to 4,258 bp, with an average length of 377 ± 227 bp. The sequencing coverage ranged from 1 to 653 with an average of 31. The distribution of contigs size and coverage are shown in Figures 1B and 1C, respectively. Contiguous sequences were further assembled into 33,307 isotigs. Isotigs are the putative transcripts constructed using the overlapping contig reads provided as input to the Newbler cDNA assembler. The size distribution of isotigs which ranged from 101 to over 4,941 bp, with an average length of 532 ± 263 bp, are shown in Figure 1D. More than 95% of the isotigs were between 101 to 1000 bp long and 50% of the assembled bases were incorporated into isotigs greater than 552 bp (N50 = 552 bp). The coverage depth for isotigs ranged from 1 to 14, with an average of 2.1 contigs assembled into each isotig. The isotigs and singletons together resulted in 409,789 unique sequences.

### Functional annotation

**Figure 2:**
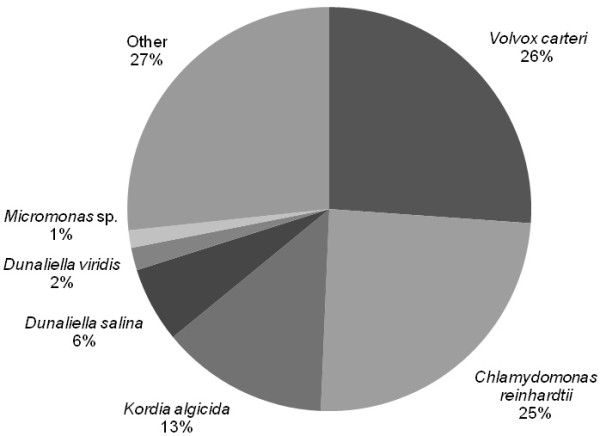
**Top-hit species distribution of BLAST matches of *D. tertiolecta *unique sequences**. Analysis of BLAST matches demonstrates the distinct microalgae character of the transcribed genes.

All unique sequences were aligned against the sequences in the National Center for Biotechnology Information (NCBI) non-redundant (nr) protein database using the BLASTx algorithm. Using an E-value threshold of 10^-6^, a total of 8,466 isotigs (25% of total isotigs), and 15,888 singleton sequences (4% of total singletons) had significant BLAST matches (Table 2). The frequency of annotated isotigs is consistent with the 20 to 40% values previously reported for *de novo *transcriptome assemblies of eukaryotes [20,21,31,32]. Sequences that did not have BLASTx matches but met quality control may still have biological significance and may be important in future, directed studies on *D. tertiolecta *metabolism. In total, 24,354 unique sequences were identified through BLAST searches. Analysis of BLAST matches demonstrated a distinct microalgae character of the transcribed genes. The top-hit species distribution of BLAST matches is shown in Figure 2. Approximately 60% of the sequences had significant matches with closely related microalgae species, predominantly *Volvox carteri *and *Chlamydomonas reinhardtii*. A phylogenetic tree inferring the evolutionary relationship between *D. tertiolecta *and these organisms is presented in the Additional file 2. *C. reinhardtii *is a model microalga with a sequenced genome and has been the focus of most physiological, molecular, and genetic studies in microalgae. *V. carteri *is a multicellular alga and a draft genome sequence has been completed for this organism. BLAST search also identified 13% of the sequences as being similar with the marine algicidal bacterium *Kordia algicida*, and the remaining 27% were related to plant species and other marine microalgae and bacteria. The similarity of BLAST results with *Kordia algicida *indicates that this microorganism might share some genetic information with *D. tertiolecta*, or be present in our samples. The latter, however, is very unlikely since we used pure cultures of *D. tertiolecta*, and constructed the cDNA libraries using poly-T primers.

**Table 2:** D. tertiolecta transcriptome annotation summary

	Number of sequences		
	
	Isotigs	Singletons	Total unique sequences
Total number of sequences	33,307	376,482	400,789
Sequences with BLAST matches	8,466	15,888	24,354
Sequences annotated with Gene Ontology (GO) terms	5,354	10,332	15,686
Sequences assigned with Enzyme Commission (EC) numbers	2,289	4,788	7,077

**Figure 3:**
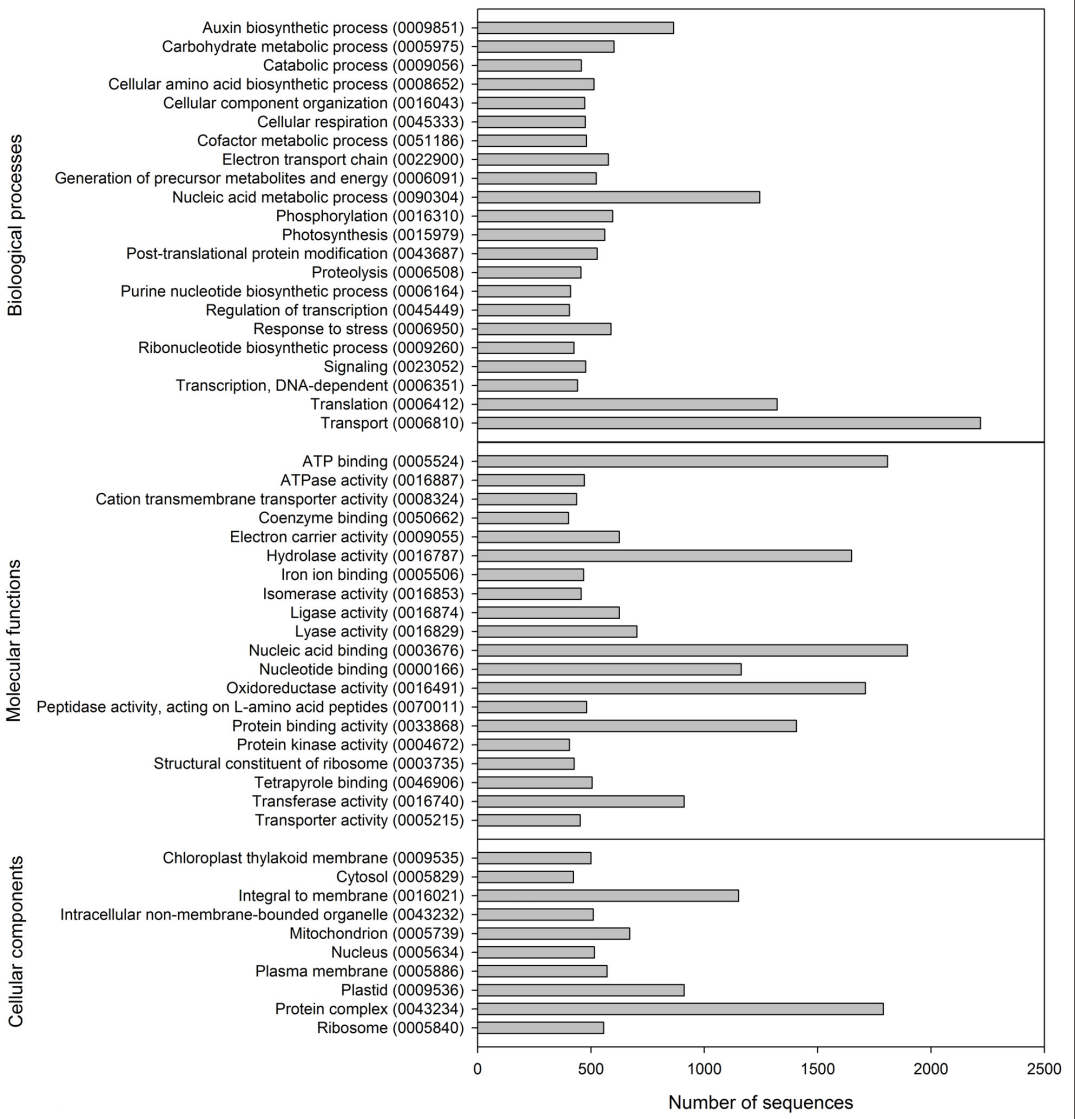
**Distribution of most abundant Gene ontology (GO) terms assigned to the *D. tertiolecta *transcriptome**. Only GO terms containing 400 sequences or more are presented. Corresponding GO IDs are presented in parentheses.

Using the Blast2Go platform [33], sequences with a BLAST match were further annotated with Gene Ontology (GO) terms and enzyme commission (EC) numbers. GO terms were assigned to a majority of isotigs (63%) and singletons (65%) (Table 2). The distribution of most abundant GO terms for biological processes, molecular functions, and cellular components is presented in Figure 3. Of the 15,686 sequences annotated with GO terms, 7,077 sequences were assigned with EC numbers (2,289 of isotigs, and 4,788 of singletons) (Table 2). To further enrich the annotation of our transcriptome library, unique sequences were assigned with KEGG orthology (KO) identifiers using KEGG Automatic Annotation Server (KAAS) [34], and subsequently mapped to BRITE functional hierarchies. The BRITE functional hierarchies linked many of the annotated sequences with biological systems such as genetic and environmental information processing, and cellular processes in *D. tertiolecta *[Additional file 3].

### Pathway classification by KEGG

To reconstruct the metabolic pathways involved in *D. tertiolecta*, annotated sequences were mapped to the Kyoto Encyclopedia of Genes and Genomes (KEGG) using the Blast2Go platform [35]. We identified transcripts coding for all enzymes related to the major metabolic pathways in *D. tertiolecta *(Table 3). The completeness of these reconstructed pathways indicates that the gene function assignments were biologically meaningful and that the EC numbers have been correctly assigned to annotated sequences. Comparative analysis of enzyme-coding sequences between *D. tertiolecta *and model organisms, *Volvox carteri*, and *Chlamydomonas reinhardtii*, using BLASTx analysis revealed relatively low homology between *D. tertiolecta *and these organisms for the enzymes described in this study (Table 4, 5, 6). These differences indicate that functional genomics and metabolic engineering of *D. tertiolecta *cannot be fully based on the sequence information obtained from model organisms, and further demonstrates the importance of annotated *D. tertiolecta *transcriptome as a genetic framework required for carrying out such studies. Because of their relevance to production of precursors for biofuel production, the metabolic pathways associated with biosynthesis and catabolism of lipids and starch were given further treatment below. A more fundamental understanding of these pathways in microalgae is required to direct efforts to enhance the microalgae-biofuel production and produce the fuel characteristics needed for commercialization.

**Table 3:** Essential metabolic pathways annotated in the *D. tertiolecta *transcriptome

Pathway	Enzymes found	Known enzymes
Photosynthetic carbon fixation (Calvin cycle)	12	13
Glycolysis/Gluconeogenesis	10	10
Pentose phosphate	5	5
Citrate cycle	10	10
Fatty acid biosynthesis	6	6
TAG biosynthesis	3	4
Starch biosynthesis	4	4

**Table 4:** Enzymes involved in fatty acid biosynthesis and metabolism identified by annotation of the *D. tertiolecta *transcriptome

Enzyme	Symbol	EC Number	Number of transcripts	^1^%Sequence alignment with corresponding enzymes in model organisms (Accession #)	
				
				*C. reinhardtii*	*V. carteri*
**Fatty acid biosynthesis**					

Biotin carboxylase	BC	6.3.4.14	3	^2^NM	46 (EFJ41621.1)
Acetyl-CoA carboxylase	ACC	6.4.1.2	7	28 (XP_001700442.1)	28 (EFJ46223.1)
malonyl-CoA-ACP transacylase	MAT	2.3.1.39	4	NM	62 (EFJ49352.1)
Beta-ketoacyl-ACP synthase I	KAS I	2.3.1.41	7	NM	NM
Beta-ketoacyl-ACP synthase II	KAS II	2.3.1.179	12	NM	NM
Beta-ketoacyl-ACP synthase III	KAS III	2.3.1.180	1	NM	NM
Beta-ketoacyl-ACP reductase	KAR	1.1.1.100	7	63 (XP_001703473.1)	61 (EFJ52014.1)
3R-hydroxyacyl-ACP dehydrase	HAD	4.2.1.-	2	NM	NM
Enoyl-ACP reductase (NADH)	EAR	1.3.1.9	5	NM	NM
Oleoyl-ACP thioesterase	OAT	3.1.2.14	1	NM	NM
Acyl-ACP thioesterase A	FatA	3.1.2.14, 3.1.2.-	1	NM	NM
Acyl-ACP thioesterase B	FatB	3.1.2.14, 3.1.2.-	0	NM	NM

**Fatty acid desaturation**					

Δ^9 ^Acyl-ACP desaturase	AAD	1.14.19.2	5	76 (XP_001691597.1)	76 (EFJ49192.1)
Δ^12^(ω^6^)-Desaturase	Δ^12^D	1.14.19.6	3	54 (XP_001691669.1), 47 XP_001693068.1)	54 (XP_002955859.1), 47 (XP_002949932.1)
Δ^15^(ω^3^)-Desaturase	Δ^15-^D	1.14.19.-	2	NM	NM

**Fatty acid elongation**					

3-Hydroxyacyl-CoA dehydrogenase	CHAD	1.1.1.35	9	NM	NM
Long-chain-3-hydroxyacyl-CoA dehydrogenase	LCHAD	1.1.1.211	2	NM	NM
Enoyl-CoA hydratase	ECH	4.2.1.17	16	NM	70 (EFJ49010.1)
Trans-2-enoyl-CoA reductase (NADPH)	TER	1.3.1.38	3	90 (XP_001690095.1)	NM
Palmitoyl-CoA hydrolase	PCH	3.1.2.22	1	19 (XP_001698944.1)	19 (EFJ40725.1)

**Fatty acid catabolism**					

Long-chain acyl-CoA synthetase	ACSL	6.2.1.3	3	77 (XP_001693692.1)	77 (EFJ51208.1)
Acyl-CoA oxidase	AOx	1.3.3.6	2	39 (XP_001699193.1)	NM
Acyl-CoA dehydrogenase	ACADM	1.3.99.3	16	45 (XP_001699193.1), 84 (XP_001693484.1), 73 (XP_001695945.1)	NM
Enoyl-CoA hydratase	ECH	4.2.1.17	16	NM	70 (EFJ49010.1)
Long-chain 3-hydroxyacyl-CoA dehydrogenase	LCHAD	1.1.1.211	2	NM	NM
Acetyl-CoA acyltransferase	ACAT	2.3.1.16	1	NM	NM
3-hydroxyacyl-CoA dehydrogenase	CHAD	1.1.1.35	9	NM	NM
Acetyl-CoA C-acetyltransferase	thiL	2.3.1.9	6	39 (XP_001694888.1)	34 (EFJ47048.1), 34 (EFJ39622.1), 34 (EFJ46961.1)
3-hydroxybutyryl-CoA epimerase		5.1.2.3	8	NM	NM
Enoyl-CoA isomerase	ECI	5.3.3.8	8	NM	NM
Alcohol dehydrogenase	ADH	1.1.1.1	3	80 (XP_001693934.1)	53 (EFJ45245.1)
Aldehyde dehydrogenase (NAD^+^)	ALDH	1.2.1.3	3	24 (XP_001696928.1)	21 (EFJ40853.1)
Ferredoxin-NAD^+ ^reductase	FNR	1.18.1.3	1	NM	NM

^1^In cases where multiple transcripts have been aligned with the associated enzymes in the model organisms, average similarity is reported.

^2^NM denotes that the annotated transcripts did not match the sequence of corresponding enzyme in model organisms.

**Table 5:** Enzymes involved in TAG biosynthesis and catabolism identified by annotation of the *D. tertiolecta *transcriptome

Enzyme	Symbol	EC Number	Number of transcripts	^1^%Sequence alignment with corresponding enzymes in model organisms (Accession #)	
				
				*C. reinhardtii*	*V. carteri*
**TAG biosynthesis**					

Glycerol kinase	GK	2.7.1.30	16	42 (A8IYH1)	NM
Glycerol-3-phosphate *O*-acyltransferase	GPAT	2.3.1.15	0	^2^NM	NM
1-Acyl-sn-glycerol-3-phosphate *O*-acyltransferase	AGPAT	2.3.1.51	2	NM	NM
Phosphatidate phosphatase	PP	3.1.3.4	1	NM	NM
Diacylglycerol *O*-acyltransferase	DGAT	2.3.1.20	2	NM	NM
Phospholipid:diacyglycerol acyltransferase	PDAT	2.3.1.158	1	NM	NM

**TAG catabolism**				NM	NM

Triacylglycerol lipase	TAGL	3.1.1.3	2	NM	NM

^1^In cases where multiple transcripts have been aligned with the associated enzymes in the model organisms, average similarity is reported.

^2^NM denotes that the annotated transcripts did not match the sequence of corresponding enzyme in model organisms.

**Table 6:** Enzymes involved in starch biosynthesis and metabolism identified by annotation of the *D. tertiolecta *transcriptome

Enzyme	Symbol	EC Number	Number of transcripts	^1^%Sequence alignment with corresponding enzymes in model organisms (Accession #)	
				
				*C. reinhardtii*	*V. carteri*
**Starch biosynthesis**					

Phosphoglucomutase	PGM	5.4.2.2	10	^2^NM	NM
ADP-glucose pyrophosphorylase	AGPase	2.7.7.27	7	49 (EDP04344.1), 84 (EDP08701.1)	NM
Starch synthase	SS	2.4.1.21	17	92 (EDP00372.1)	NM
1,4-α-Glucan branching enzyme	BE	2.4.1.18	6	87 (EDP05581.1)	82 (EFJ46288.1)
Sucrose phosphorylase	SuPase	2.4.7.1	3	NM	NM
Hexokinase	HXK	2.7.1.1	6	NM	NM

**Starch catabolism**					

α-Amylase	(α-AMY)	3.2.1.1	9	98 (EDP00963.1)	NM
oligo-1,6-Glucosidase	(O1, 6G)	3.2.1.10	1	NM	NM
Starch phosphorylase	(SPase)	2.4.1.1	10	43 (EDP02951.1), 78 (EDO98385.1)	NM

^1^In cases where multiple transcripts have been aligned with the associated enzymes in the model organisms, average similarity is reported.

^2^NM denotes that the annotated transcripts did not match the sequence of corresponding enzyme in model organisms.

### Fatty acid biosynthesis

Interest in microalgae as a potential feedstock for the production of biofuels and other valuable biomaterials is rooted in their ability to rapidly accumulate significant amounts of neutral lipids [36]. Under optimal conditions, microalgae synthesize fatty acids primarily for esterification into polar glycerol-based membrane lipids that consist of glycosylglycerides and phosphoglycerides, whereas under environmental stress conditions, many microalgae accumulate neutral triacylglycrols (TAGs) [17]. Although global fatty acid biosynthesis pathways are known in eukaryotes [37], biosynthesis and regulation of fatty acids in microalgae are not well studied.

**Figure 4:**
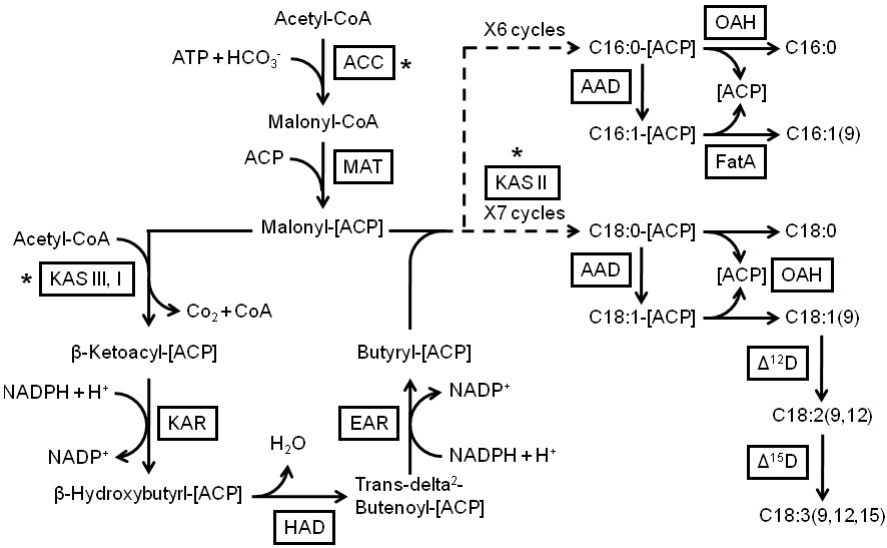
**Fatty acid biosynthesis pathway reconstructed based on the *de novo *assembly and annotation of *D. tertiolecta *transcriptome**. Identified enzymes are shown in boxes and include: ACC, acetyl-CoA carboxylase (EC: 6.4.1.2); MAT, malonyl-CoA ACP transacylase (EC: 2.3.1.39); KAS, beta-ketoacyl-ACP synthase (KAS I, EC: 2.3.1.41; KASII, EC: 2.3.1.179; KAS III, EC: 2.3.1.180); KAR, beta-ketoacyl-ACP reductase (EC: 1.1.1.100); HAD, beta-hydroxyacyl-ACP dehydrase (EC: 4.2.1.-); EAR, enoyl-ACP reductase (EC: 1.3.1.9); AAD, acyl-ACP desaturase (EC: 1.14.19.2); OAH, oleoyl-ACP hydrolase (EC: 3.1.2.14); FatA, Acyl-ACP thioesterase A (EC: 3.1.2.-); Δ^12^D, Δ^12^(ω^6^)-desaturase (EC: 1.4.19.6); Δ^15^D, and Δ^15^(ω^3^)-desaturase (EC: 1.4.19.-). The fatty acid biosynthesis pathway in *D. tertiolecta *produces saturated, C16:0 (palmitic acid) and C18:0 (stearic acid), and unsaturated fatty acids C18:1 (9) (oleic acid), C18:2 (9,12) (linoleic acid), and C18:3(9,12,15) (α-linolenic acid). Key enzymes are shown with an asterisk next to the boxes.

Based on the functional annotation of the transcriptome, we have successfully identified the genes encoding for key enzymes involved in the biosynthesis and catabolism of fatty acids in *D. tertiolecta*. Table 4 lists the enzymes involved in fatty acid biosynthesis in *D. tertiolecta*. On average, four transcripts were identified per enzyme. The reconstructed pathway based on these identified enzymes is depicted in Figure 4. The fatty acid biosynthesis pathway in microalgae primarily occurs in the chloroplast, and produces saturated C16:0 (palmitic acid) and C18:0-ACP (acyl carrier protein). These fatty acids can then be used as precursors for the synthesis of neutral lipids (mainly TAGs). Fatty acid biosynthesis in *D. tertiolecta *starts with acetyl-CoA carboxylase (ACC, EC: 6.4.1.2), which catalyzes the formation of malonyl-CoA from acetyl-CoA and bicarbonate. Next, malonyl-CoA ACP transacylase (MAT, EC: 2.3.1.39) catalyzes the transfer of malonyl-CoA to malonyl-ACP, the carbon donor for subsequent elongation reactions. During the elongation, the malonyl group of malonyl-ACP participates in a series of condensation reactions with acyl ACP (or acetyl-CoA) acceptors that are catalyzed by the multiple isoforms of the condensing enzyme, ketoacyl-ACP synthase (KAS) (Table 4). The first condensation reaction is catalyzed by beta-ketoacyl-ACP synthase III (KAS III, EC: 2.3.1.180) and results in a four-carbon product. The other condensing enzyme, beta-ketoacyl-ACP synthase I (KAS I, EC: 2.3.1.41), produces varying chain lengths (6 to 16 carbons). Finally, beta-ketoacyl-ACP synthase II (KASII, EC: 2.3.1.179) is responsible for the elongation of C16:0-ACP to C18:0-ACP. The initial product of each condensation reaction is a beta-ketoacyl-ACP. Three additional reactions (reduction, dehydration, and another reduction) occur after each condensation. To form a saturated fatty acid, the beta-ketoacyl ACP product is reduced by the enzyme beta-ketoacyl-ACP reductase (KAR, EC: 1.1.1.100), dehydrated by 3R-hydroxyacyl-ACP dehydrase (HAD, EC: 4.2.1.-), and then reduced by enoyl-ACP reductase (EAR, EC: 1.3.1.9). These four reactions lead to a lengthening of the precursor fatty acid by two carbons for every round of cycle. To produce 16 or 18 carbon fatty acids, this cycle is repeated for 6 or 7 times respectively (Figure 4).

For the synthesis of unsaturated fatty acids in plastid, a double bond is introduced to the acyl group esterified to ACP via the enzyme acyl-ACP desaturase (AAD, EC: 1.14.19.2). The elongation of fatty acids in the chloroplast is terminated when the acyl group is removed from ACP by acyl-ACP thioesterase enzymes, oleoyl-ACP hydrolase (OAT, EC: 3.1.2.14), or when acyl-ACP thioesterase A (FatA) hydrolyze the acyl-ACP and releases the free fatty acid, or when acyl transferases in the chloroplast transfers the fatty acid directly from ACP to glycerol-3-phosphate or monoacylglycerol-3-phosphate. The final fatty acid composition is determined by the activities of enzymes that use these acyl-ACPs at the termination phase of fatty acid synthesis. We have also identified desaturation enzymes Δ^12^(ω^6^)-desaturase (Δ^12^D, EC: 1.4.19.6), which desaturates oleic acid (18:1n-9) to form linoleic acid (18:2n-6), and Δ^15^(ω^3^)-desaturase (Δ^15^D, EC: 1.4.19.-), which further desaturates linoleic acid to form α-linolenic acid (18:3n-3). The annotation of *D. tertiolecta *transcriptome did not identify any genes encoding enzymes involved in further desaturation and elongation of linoleic and linolenic acids that could result in production of longer chain polyunsaturated fatty acids. The lack of identification of these enzymes is consistent with the profile of fatty acids produced by *D. tertiolecta *[38,39].

In addition to synthesis, all the enzymes involved in fatty acid catabolism (β-oxidation pathway) of *D. tertiolecta *were successfully identified and are presented in Table 4 (coded by six transcripts on average). The fatty acid catabolism pathway is provided in Additional file 4. The β-oxidation pathway in microalgae involves four enzymes: acyl-coA oxidase (AOx, EC: 1.3.3.6); enoyl-CoA hydratase (ECH, EC: 4.2.1.17); 3-hydroxyacyl-CoA dehydrogenase (CHAD, EC: 1.1.1.35); and acetyl-CoA acyltransferase (ACAT, EC: 2.3.1.16), which collectively catalyze the cleavage of two carbons from the acyl chain during each cycle. The resulting acetyl-CoA is then used to produce energy for the cell via the citrate cycle.

The *D. tertiolecta *transcriptome presented here contains most of the enzymes required for the biosynthesis, elongation, and metabolism of fatty acids (Table 4), and the subsequent reconstructed pathways are consistent with those proposed for model microalgae *C. reinhardtii *[17], and plants in general [37,40-42]. These findings contribute to the biochemical and molecular information needed for metabolic engineering of fatty acid synthesis in microalgae. The most commonly stated strategy is the overexpression of ACC, the rate-limiting step in fatty-acid biosynthesis [43]. The condensing enzymes that are identified in this study are also potential targets for improving fatty acid biosynthesis. For example, Verwoert et al. has shown that the over-expression of KAS III in *Brassica *seeds alter the composition of fatty acids but does not change the per cell quantity [44]. A final example approach for per cell fatty acid enrichment in microalgae is to block lipid catabolism [45], which could then result in increased lipid storage.

### Triacylglycerols (TAG) biosynthesis and catabolism

**Figure 5:**
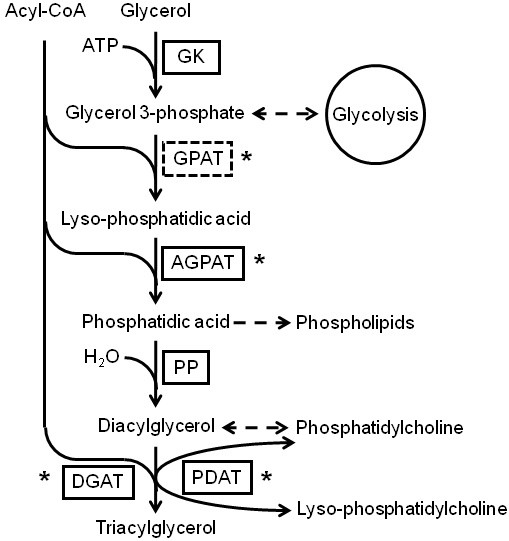
**Triacylglycerol biosynthesis pathway reconstructed based on the *de novo *assembly and annotation of *D. tertiolecta *transcriptome**. Identified and unidentified enzymes are shown in solid and dashed boxes, respectively, and include: GK, glycerol kinase (EC: 2.7.1.30); GPAT, glycerol-3-phosphate *O*-acyltransferase (EC: 2.3.1.15); AGPAT, 1-acyl-sn-glycerol-3-phosphate *O*-acyltransferase (EC:2.3.1.51); PP, phosphatidate phosphatase (EC: 3.1.3.4); DGAT, diacylglycerol *O*-acyltransferase (EC: 2.3.1.20); and PDAT, phopholipid:diacyglycerol acyltransferase (EC 2.3.1.158). Key enzymes are shown with an asterisk next to the boxes, and dashed arrows denote reaction(s) in which the enzymes are not shown.

Some species of microalgae are capable of producing and accumulating high amounts of neutral storage lipids, mainly TAGs, under stress-inducing conditions. TAGs can serve as precursors for production of biodiesel and other bio-based products such as plastics, cosmetics, and surfactants [36]. Although the global pathways for TAG biosynthesis are known, the existing knowledge on the pathways and enzymes involved in TAG synthesis in microalgae is limited [46,47]. Based on the KEGG pathway assignment of the functionally annotated sequences, transcripts coding for most of the enzymes involved in TAG biosynthesis were found in *D. tertiolecta*. These enzymes are presented in Table 5, and the suggested pathway for TAG synthesis in *D. tertiolecta *is shown in Figure 5. The precursors for TAG biosynthesis are glycerol-3-phosphate (G-3-P), and acyl-CoA. G-3-P is produced by the catabolism of glucose (glycolysis) or to a lesser extent by the action of the enzyme glycerol kinase (GK, EC: 2.7.1.30) on free glycerol. We identified 16 transcripts in *D. tertiolecta *transcriptome library coding for this enzyme. The acyl-CoA, on the other hand, is generated via esterification of fatty acids produced in the chloroplast to coenzyme A. The first two steps of TAG biosynthesis involve sequential esterification of acyl chains from acyl-CoA to positions 1 and 2 of G-3-P, resulting in the formation of phosphatidic acid (PA), which is a key intermediate in the biosynthesis of all glycerolipids. These steps are catalyzed by glycerol-3-phosphate *O*-acyltransferase (GPAT, EC: 2.3.1.15) and 1-acyl-sn-glycerol-3-phosphate *O*-acyltransferase (AGPAT, EC: 2.3.1.51), respectively. Genes encoding for AGPAT were identified in the *D. tertiolecta *transcriptome library, however, genes encoding for GPAT were not identified. The next reaction in the pathway is catalyzed by the enzyme phosphatidate phosphatase (PP, EC: 3.1.3.4), which removes the phosphate group from PA and produces the diacylglycerol (DAG). One transcript was annotated as coding for this enzyme in the *D. tertiolecta *transcriptome library. The DAG is an essential intermediate in the biosynthesis of phosphatidylcholine (PC) and phosphatidylethanolamine (PE). Finally, the resultant DAG is acylated, at the position 3 using an acyl donor to form the TAG. We identified transcripts coding for two distinct enzymes that catalyze this reaction. These enzymes differ in their source of acyl-donor. The first enzyme is diacylglycerol *O*-acyltransferase (DGAT, EC: 2.3.1.20), which uses acyl-CoA as an acyl-donor and is believed to be the main pathway for biosynthesis of TAG [17,48]. The second enzyme is phopholipid:diacyglycerol acyltransferase (PDAT, EC: 2.3.1.158), and uses phospholipids as acyl donors. This acyl-CoA independent route for TAG biosynthesis had been previously reported in some plants and yeast [49], and the gene encoding for PDAT has been identified in the sequenced genome of *C. reinhardtii *[46]. Identification of PDAT in *D. tertiolecta *provides further evidence that some microalgae might have the potential to channel the fatty acids incorporated in membrane lipids (e.g. PC), into the TAG synthesis. The identification of this alternative route for the flux of fatty acids into and out of TAG synthesis can also provide insight into the connection between rapid degradation of membrane lipids with concurrent accumulation of TAGs when microalgae are exposed to stress conditions [17]. The functional importance of PDAT in TAG biosynthesis, however, remains to be determined via gene-knockout experiments and analysis of lipid profiles.

Additionally, we identified transcripts coding for enzymes related to catabolism of TAG. The complete breakdown of TAG takes place in two stages. First, hydrolysis of ester bonds that link fatty acyl chains to the glycerol backbone is catalyzed by lipases. We found two transcripts in the *D. tertiolecta *transcriptome coding for triacylglycerol lipase (TAGL, EC: 3.1.1.3), which releases fatty acids from DAG and TAG. In the second stage, the fatty acids that are liberated may be further broken down by oxidation or follow other metabolic pathways including re-esterification with glycerol to from new acylglycerols [42]. Suppression of enzymes involved in TAG degradation, such as TAGL, could potentially increase the TAG content. Though this approach has previously resulted in elevated levels of TAG in transgenic plants, it severely limits plant growth [50]. Another potential approach includes manipulation of acyltransferases enzymes, as they are the key determinant of content and acyl composition of glycerolipids [51-54]. Identification of transcripts coding for these enzymes in *D. tertiolecta *provide the first step for attempts to genetically engineer this organism to increase the production and modify the composition of the lipids [45,46].

### Starch biosynthesis and catabolism

As the main assimilatory product of photosynthesis, some species of microalgae synthesize a significant amount of starch as storage materials in their plastids [29,55]. The accumulated starch is an attractive substrate for the production of a variety of biofuels, including ethanol, butanol, and hydrogen [56-59]. Production of biofuel from microalgae-based starch potentially overcomes the sustainability and pretreatment process disadvantages ascribed to using plant-based starch and lignocellulosic materials as ethanol feedstock [57,60]. Though the pathways associated with biosynthesis and degradation of starch are well studied in plants and the model microalgae *C. reinhardtii*, such knowledge is scarce in non-model microalgae with direct biofuel potentials.

**Figure 6:**
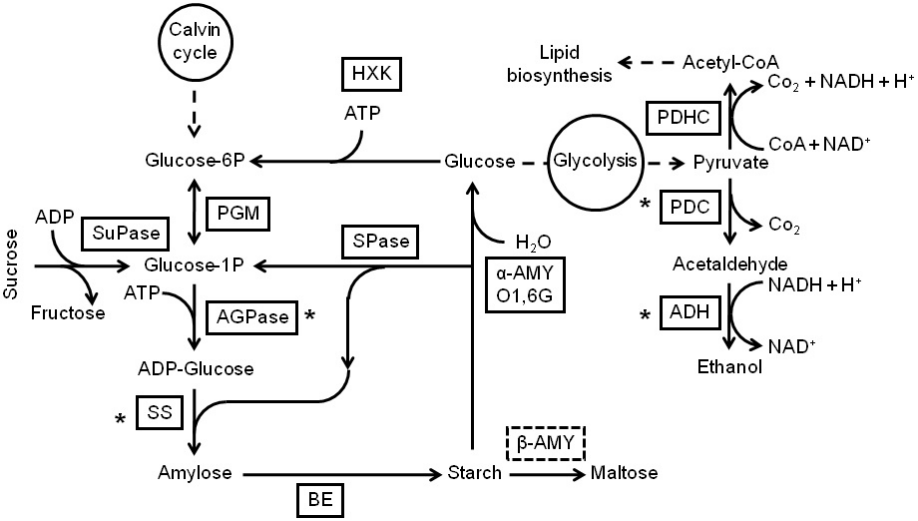
**Starch biosynthesis and catabolism, and ethanol fermentation pathways reconstructed based on the *de novo *assembly and annotation of *D. tertiolecta *transcriptome**. Identified and unidentified enzymes are shown in solid and dashed boxes, respectively. Enzymes involved in starch biosynthesis include: PGM, phosphoglucomutase (EC: 5.4.2.2); SuPase, sucrose phosphorylase (EC: 2.4.7.1); AGPase, ADP-glucose pyrophosphorylase (EC: 2.7.7.27); SS, starch synthase (EC: 2.4.1.21); BE, α-1,4-glucan branching enzyme (EC: 2.4.1.18); and HXK, hexokinase (2.7.1.1). Enzymes involved in starch catabolism include: α-AMY, α-amylase (EC: 3.2.1.1); O1, 6G, oligo-1,6-glucosidase (EC: 3.2.1.10); β-AMY, β-amylase (EC: 3.2.1.2); and SPase, starch phosphorylase (EC: 2.4.1.1). Enzymes involved in ethanol fermentation via pyruvate include: PDC, pyruvate decarboxylase (EC: 4.1.1.1); and ADH, alcohol dehydrogenase (EC: 1.1.1.1). Enzyme PDHC, Pyruvate dehydrogenase complex (EC 1.2.4.1, 2.3.1.12, 1.8.4.1), transforms pyruvate into acetyl-CoA which may then be used in the lipid biosynthesis pathway. Key enzymes are shown with an asterisk next to the boxes, and dashed arrows denote reaction(s) in which the enzymes are not shown.

Based on the KEGG pathway assignments, we identified numerous transcripts coding for enzymes involved in the biosynthesis and catabolism of starch in *D. tertiolecta *(Table 6). The pathway of starch synthesis in *D. tertiolecta *involves the enzymes phosphoglucomutase (PGM, EC: 5.4.2.2), which generates glucose-1-phosphate (Glc-1-P) from glucose-6-phosphate (Glc-6-P), and ADP-glucose pyrophosphorylase (AGPase, EC: 2.7.7.27), which uses Glc-1-P and ATP to generate ADPGlc and inorganic pyrophosphate. ADPGlc is the substrate for starch synthase (SS, EC: 2.4.1.21), which generates ADP and an amylose, a linear chain of glucose residues connected by α-1,4-glycosidic bonds. The final step in the pathway involves α-1,4-glucan branching enzyme (BE, EC: 2.4.1.18), which catalyzes the formation of α-1,6-glycosidic bonds in the elongated glucans that comprise starch. Based on the identified enzymes from the retrieved transcripts, biosynthesis of starch from Glc-6-P in *D. tertiolecta *resembles the classical pathway of starch synthesis proposed in plants and *C. reinhardtii *[61]. We also identified transcripts that code for sucrose phosphorylase (SuPase, EC: 2.4.7.1), which generates G-1-P from sucrose as an alternative source of substrate for starch synthesis. This alternative pathway is consistent with the newly proposed model for starch synthesis in *Arabidopsis *[62]. Experimental research (e.g. gene knock-out analysis), however, is required to support the role of SuPase in starch biosynthesis in *D. tertiolecta*. Figure 6 shows the reconstructed pathway for biosynthesis of starch in *D. tertiolecta*. Although our results do not allow speculation on the cellular location of starch biosynthesis in *D. tertiolecta*, previous studies on *D. marina *starch pathway suggest that it is exclusively intraplastidic [63], which is consistent with starch synthesis and storage in the *Cryptophyceae *class of microalgae [64].

The starch biosynthesis pathway was well represented in our library as indicated by the number of transcripts assigned to each enzyme (on average 9 transcripts per enzyme). All of known enzymes in the starch synthesis pathway presented were identified (Table 6). Genetic manipulation of key enzymes, mainly AGPase and to less extent SS, involved in the process has been tried to increase starch contents in crop plants. Much of the efforts have been focused on AGPase as this enzyme catalyses a rate-limiting step in the biosynthesis of starch, and thus increase in its activity could lead to increased rate of starch synthesis [65].

The starch accumulated in green microalgae is considered to be mostly utilized in the respiration. We identified two distinct pathways, namely hydrolytic and phosphorolytic, involved in starch catabolism in *D. tertiolecta *(Figure 6). Twenty transcripts in our library were annotated as coding for enzymes involved in these pathways (Table 6). The enzymes related to the hydrolytic pathway include α-amylase (α-AMY, EC: 3.2.1.1), and oligo-1,6-glucosidase (O1, 6G, EC: 3.2.1.10). These two enzymes catalyze the hydrolysis of starch to oligosaccharides (i.e. dextrin) and further to α-D-glucose, respectively. The released α-D-glucose maybe further degraded through glycolysis or be phosphorylated by hexokinase (HXK, 2.7.1.1), for reentry into the starch synthesis pathway. We did not identify transcripts that code for β-amylase (β-AMY, EC: 3.2.1.2), which degrades starch into maltose. The phosphorolytic degradation of starch in *D. tertiolecta *may involve starch phosphorylase (SPase, EC: 2.4.1.1), which mediates the transfer of glucose from the non-reducing end of an α-1,4-linked glucan to orthophosphate and generates G-1-P and a shorter glucan. Further investigations are warranted to determine the relative importance of these pathways in *D. tertiolecta*.

### Pathways interactions, carbon partitioning and source-sink relationships

The metabolic pathways associated with biosynthesis and degradation of energy-rich molecules are closely linked. Starch catabolism provides the metabolites for biosynthesis of other energy rich products. Our KEGG pathway assignments revealed that *D. tertiolecta *has the genetic potential to link starch metabolism to ethanol fermentation through the glycolysis pathway (Figure 6) (Also see Additional files 5 and 6 for the pathway map and the complete set of identified enzymes involved in glycolysis, respectively). We identified transcripts coding for enzymes that catalyze the synthesis of ethanol from the intermediate metabolite, pyruvate in *D. tertiolecta*. These enzymes include pyruvate decarboxylase (PDC, EC: 4.1.1.1), which generates acetaldehyde and CO_2 _from pyruvate, and alcohol dehydrogenase (ADH, EC: 1.1.1.1), which uses acetaldehyde and NADH + H^+ ^to generate ethanol. Although ethanol production has been previously observed in marine microalgae [66], no reports exist in *D. tertiolecta *and existence of ethanol fermentation pathway raises the potential that this organism could be engineered to be an efficient converter of solar energy into ethanol.

Additionally, biosynthesis of starch can direct the flow of metabolites away from lipid biosynthesis and conversely starch degradation provides the metabolites for production of energy rich molecules (i.e, lipids, and ethanol). We identified 20 transcripts that code for a pyruvate dehydrogenase complex (PDHC) (EC: 1.2.4.1, 2.3.1.12, 1.8.1.4) that transforms pyruvate into acetyl-CoA through pyruvate decarboxylation. Acetyl-CoA may then be used in the fatty acid synthesis pathway. A blockage of starch synthesis has been shown to increase the accumulation of lipids in several starchless mutants of microalgae [30,67]. Disruption of genes related to starch degradation or over expression of genes involved in starch synthesis have successfully resulted in increased starch content in microalgae and *Arabidopsis thaliana *[45,65].

Concerted production and accumulation of energy rich molecule in microalgae also depends upon the integration of precursor supplying pathways (i.e. sources) with synthesizing machineries (i.e. sinks). The accumulation of large quantities of lipids in microalgae requires a continuous supply of acetyl-CoA and NADPH. The pathways supplying these precursors lie outside of the fatty acid synthetic machinery, and it has been suggested that they are unique to oleaginous microorganisms [68]. The key supplier of acetyl-CoA for fatty acid synthesis in oleaginous microorganisms is considered to be ATP:citrate lyase (ACL, EC: 2.3.3.8), which catalyzes the formation of acetyl-CoA and oxaloacetate by cleaving citrate using an ATP molecule [68]. The formation of NADPH as an essential reductant for fatty acid synthesis has been mainly attributed to malate dehydrogenase (MDH, EC: 1.1.1.40), which uses malate and NADP^+ ^to generate pyruvate, CO_2 _and NADPH [68]. Citrate and malate are intermediates of tricarboxylic acid (TCA) cycle and pyruvate metabolism, respectively. Interestingly, we identified numerous sequences, 76 and 11, in our transcriptome library coding for ACL and MDH, respectively. The integration of these enzymes with fatty acid biosynthesizing machinery ensures the direct flow of acetyl-CoA into fatty acids, which are then used as precursors of TAG synthesis. Genetic manipulations that increase the availability of precursors for fatty acid and starch synthesis, through up-regulation/over-expression of related genes identified here, could be promising approaches to increase the yield of biofuel precursors in microalgae.

## Conclusions

This study presents the first next-generation sequencing effort and transcriptome annotation of a non-model marine microalgae that is relevant for biofuel production. Genes encoding key enzymes have been successfully identified and metabolic pathways involved in biosynthesis and catabolism of fatty acids, TAG, and starch in *D. tertiolecta *have been reconstructed. Identification of these genes and pathways is in agreement with the empirically observed capability of *D. tertiolecta *to synthesize and accumulate energy rich molecules, and adds to the current knowledge on the molecular biology and biochemistry of their production in microalgae. By providing insight into the mechanisms underpinning these metabolic processes, results can be used to direct efforts to genetically manipulate this organism to enhance the production of feedstock for commercial microalgae-biofuels.

The accumulation of biofuel precursors and discovery of genes associated with their biosynthesis and metabolism in *D. tertiolecta *is intriguing and worthy of further investigation. The sequences and pathways produced here present the genetic framework required for further studies. Quantitative transcriptomics in concert with physiological and biochemical analysis in *D. tertiolecta *under conditions that stimulate production and accumulation of biofuel precursors are needed to provide insight into the ways these pathways are regulated and linked.

## Methods

### *D. tertiolecta *culturing, harvesting and RNA extraction

*D. tertiolecta *(UTEX LB 999) was obtained from the Culture Collection of Algae at the University of Texas. Cells were cultured in 1 L flasks filled with 750 ml of Erdschreiber's medium (UTEX), modified to have different concentrations of nitrogen and salinity. Reactors were operated at room temperature in batch mode and exposed to fluorescent light (32 Watts, Cool White) at a photosynthetic photon flux density (PPFD) of 135 μmol-photon m^-2 ^s^-1^, with a 14/10 h light/dark cycle. Gas flow rate was 200 ml min^-1 ^of house air and controlled using a mass flow controller (Cole-Parmer Instrument Company, IL, USA). The air stream was passed over activated carbon and filtered through a 0.2 μm filter before being flushed into the reactors. All cultures were mixed by an orbital shaker at 200 rpm.

Cells were cultured under various growth conditions and at different phases of the growth cycle. These various growth conditions and phases were chosen to stimulate production and accumulation of lipids or starch, to induce expression of genes involved with lipids or starch biosynthesis, and to maximize the diversity of expressed genes [23,28-30,69]. The growth conditions included: nitrogen limited cultures (10 mg L^-1^, N) with salinity levels similar to that of seawater (i.e. 0.5 M NaCl) harvested during stationary phase (sample A), nitrogen sufficient cultures (100 mg L^-1^, N) with salinity of 0.5 M NaCl, harvested during exponential growth (sample B), and stationary (samples C) phases, and salt-stressed cultures (100 mg L^-1^, N) with elevated salt concentration (1.5 M NaCl) harvested during the stationary phase (sample D). Cell growth was monitored in duplicate reactors under each condition by measuring the changes of optical density in the culture medium at 730 nm (OD_730_), using a spectrophotometer (HP 8453, Hewlett Packard, CA, USA). Cells were harvested by centrifugation (RC-6 Plus, ThermoScientific, DE, USA), at 17,000 *g*, for 5 min, at 4°C. The supernatant was discarded and cell pellets were immediately frozen in liquid nitrogen and stored at -80°C until further analysis.

Total RNA from cells was extracted and purified using RNeasy Plant Mini Kit (Qiagen, CA, USA) with the following modifications for cell lysis. Cell pellets were re-suspended in buffer RLT (1 × 10^5 ^cells μl^-1^), transferred to a 2-ml screw cap tube containing 300 mg of glass beads (0.5 mm, baked at 500°C for 4 h), and lysed and homogenized by agitation in a bead-beater (Mini-Beadbeater-16, BioSpec Products, OK, USA) at the maximum speed (3450 oscillations/min) for 40 s. Bead-beating was repeated four times for each sample. The residual genomic DNA contamination was removed during the RNA cleanup using the optional on-column DNase I digestion as instructed by the manufacturer (Qiagen, CA, USA). The integrity of the purified total RNA was assessed using formaldehyde agarose gel electrophoresis, and RNA quantity was determined by NanoDrop spectrophotometer measurements (ThermoScientific, DE, USA). Because microalgae have high levels of pigments, polysaccharides, and glycolproteins that could interfere with cDNA synthesis and are difficult to remove using spin-column purification methods, total RNA was further purified using lithium-chloride precipitation as previously described [21].

### Synthesis of cDNA and library construction

Synthesis of full-length double-stranded cDNA (ds-cDNA) from total RNA was performed using SMARTer PCR cDNA Synthesis Kit (Clontech, CA, USA) according to the manufacturer's instructions, with the exception of using a modified CDS primer (5'-AAGCAGTGGTATCAACGCAGAGTACGTGCAGTTTTTTTTTTTTTTTTVN-3'). This modified primer included a recognition site (GTGCAG), on the 5' end of the poly T tail for restriction enzyme *Bsg*I. This restriction site was then used to eliminate the presence of poly (A:T) tail in the cDNA samples. These homopolymers could result in too strong of a light signal and thus produce sequencing reads of low quality when using the Genome Sequencer FLX with Titanium reagents.

Full-length cDNA templates were then amplified by long-distance PCR using the Advantage 2 PCR Kit (Clontech, CA, USA). To ensure that the PCR products were not over amplified, the optimal number of PCR cycles was determined according to manufacturer's guidelines. The PCR reactions were then chased [70] to maximize the quantities of fully double-stranded cDNA products and quality was verified by agarose gel electrophoresis. Replicate PCR reactions were performed for each library then pooled and purified using the QIAquick PCR Purification kit (Qiagen, CA, USA). The amplified cDNA libraries were quantified using NanoDrop (ThermoScientific, DE, USA), and equal amounts of PCR products from sample libraries B, C, and D were pooled to construct a new library "P" which was used along with library A for normalization.

To enhance gene discovery, the proportion of transcripts (i.e. expressed genes) that are highly abundant in the sample were reduced before sequencing. Equal amount of samples (1.6 μg) from cDNA libraries A and P were normalized using the Trimmer cDNA Normalization Kit (Evrogen, Moscow, Russia), and re-amplified using the 5' PCR primer II A (SMARTer PCR cDNA Synthesis Kit), according to the manufacturer's instructions. The normalized cDNA samples were then purified as described above (QIAquick PCR Purification Kit), and digested using the *Bsg*I enzyme (New England BioLabs, MA, USA) to remove the residual poly (A:T) tails. Following digestion, an aliquot of restriction digest solution was evaluated on 10% TBE polyacrylamide gels (Invitrogen, CA, USA), to verify that the appropriate fragment size (49 bp) had been cleaved. Finally, digested, normalized cDNA libraries were purified (QIAquick PCR Purification Kit), and the quality of final samples was verified using agarose gel electrophoresis.

### 454 sequencing

Sequencing of cDNA samples was performed by Roche-454 Life Sciences (Branford, CT, USA) using the Genome Sequencer FLX with Titanium Chemistry. Each of the A and P samples was sequenced on the half of a PicoTiter Plate according to the manufacturer's instructions (Roche, IN, USA). All sequencing reads were deposited into the Short Read Archive (SRA) of the National Center for Biotechnology Information (NCBI), and can be accessed under the accession number SRA023642.

### Sequence analysis, and assembly

Sequencing data obtained from samples A and P were pooled and subjected to a publicly available sequence cleaning and validation software, SeqClean [71], to account for size-selection, overall low complexity analysis, and to remove poly (A:T) regions, and adapters. In addition, a comprehensive ribosomal RNA database, Silva [72], containing regularly updated, high quality sequences of eukaryotic rRNAs were incorporated into the cleaning pipeline of the SeqClean to remove ribosomal RNA sequences. Following the sequence trimming and size selection (>100 bp), the reads were assembled using the Newbler v2.3 provided by Roche-454 Life Sciences (Branford, CT). Assembly parameters were used as default values with minimum quality score of 20, and overlap identity as 90% over 40 bp length to detect pairwise alignments. Assembly computations were duplicated to make sure that the results were reproducible.

### Unique sequence mapping, functional annotation, and pathway assignments

Following the assembly, unique transcripts (isotigs) and singletons were compared to NCBI's non-redundant (nr) database using BLASTx algorithm [73], with a cut-off E-value of ≤ 10^-6^. Resulting top 10 blast hits were fed into publicly available Blast2GO software (v.2.4.4) [33] in order to retrieve associated gene ontology (GO) terms describing biological processes, molecular functions, and cellular components [74]. By using specific gene identifiers and accession numbers, Blast2GO produces all GO annotations as well as corresponding enzyme commission numbers (EC) for sequences with an E-value equal to or less than 10^-6^. To compare the enzyme-coding genes identified in this work with those identified in the model microalgae with sequenced genome, we used BLASTx algorithm with an E-value threshold of 10^-6 ^to align the transcript sequences annotated as enzymes related to the production of biofuel precursors against the sequences of associated enzymes in *Volvox carteri*, and *Chlamydomonas reinhardtii*.

To determine metabolic pathways, Kyoto Encyclopedia of Genes and Genomes (KEGG) mapping was used [35]. The sequences with corresponding ECs obtained from Blast2GO were mapped to the KEGG metabolic pathway database. To further enrich the pathway annotation and to identify the BRITE functional hierarchies, sequences were also submitted to the KEGG Automatic Annotation Server (KAAS) [34], and the single-directional best hit information method was selected. KAAS annotates every submitted sequence with KEGG orthology (KO) identifiers, which represents an ortholog group of genes directly linked to an object in the KEGG pathways and BRITE functional hierarchy [34,75], and thus incorporates different types of relationships that exist in biological systems (i.e. genetic and environmental information processing, cellular processes, and organismal systems). The graphical KEGG Markup Language pathway editor (KGML-ED) was used to draw the fatty acid catabolism and glycolysis pathways [76]. Computationally processed assembly outputs and annotations are hosted at the corresponding author's website http://www.eng.yale.edu/peccialab/microalgae_sequences.html for public access.

## Authors' contributions

HRY and JP conceived and designed the experiments. HRY conducted the reactor-based experiments and prepared the cDNA libraries. HRY and BZH performed the bioinformatic analysis. KB was instrumental in bioinformatic analysis. HRY drafted the manuscript, constructed the tables and figures, and BZH and JP contributed to the final version. All authors have read and approved the final manuscript.

## Supplementary Material

Additional file 1 **Comparison of *D. tertiolecta *transcriptome assembly outputs obtained using Newbler v2.3 and 2.5**. Click here for file

Additional file 2 **A phylogenetic tree inferring the evolutionary relationship between *D. tertiolecta *and model microalgae *Volvox carteri*, and *Chlamydomonas reinhardtii***. The tree was generated using 18 S rRNA gene sequences of *D. tertiolecta*, *Volvox carteri*, and *Chlamydomonas reinhardtii *extracted from the NCBI database. Sequences were aligned using ClustalX and bootstrapping was performed in ClustalX with 100 iterations and values were displayed on the branch edges. The tree was visualized and published in Mega5. The distance bar represents 0.01 base changes/base. The tree was rooted with *Methanobacterium congolense *(NCBI Accession Number: AF233586.1). Click here for file

Additional file 3 **Candidate genes identified based on KEGG orthology (KO) annotation of the *D. tertiolecta *transcriptome**. Click here for file

Additional file 4 **β-oxidation pathway of *D. tertiolecta *based on the annotation of transcriptome and KEGG pathway assignment**. Click here for file

Additional file 5 **Glycolysis pathway of *D. tertiolecta *based on the annotation of transcriptome and KEGG pathway assignment**. Click here for file

Additional file 6 **Enzymes involved in glycolysis identified by annotation of the *D. tertiolecta *transcriptome**. Click here for file
