# Supplementary material for: Transcriptome sequencing and annotation of the microalgae Dunaliella tertiolecta: Pathway description and gene discovery for production of next-generation biofuels
Source: BMC Genomics. 2011 Mar 14;12:148. doi: 10.1186/1471-2164-12-148 (PMC3061936; doi:10.1186/1471-2164-12-148)
Supplement: Additional file 1 — Comparison of D. tertiolecta transcriptome assembly outputs obtained using Newbler v2.3 and 2.5. [file 1471-2164-12-148-S1.DOC]

**Additional file 1:** **Comparison of *D. tertiolecta* transcriptome assembly outputs obtained using Newbler v2.3 and v2.5.**

| Assembly outputs | v2.3 | v2.5 |
| --- | --- | --- |
| Contigs |  |  |
| Reads assembled as contigs | 609,149 | 662,900 |
| Number of contigs | 34,301 | 29,942 |
| Average length of contigs | 377 bp | 382 bp |
| Range of contigs length | 86-4,258 bp | 86-2,840 bp |
| Depth on contigs | 31 | 28 |
|  |  |  |
| Isotigs |  |  |
| Number of isotigs | 33,307 | 29,451 |
| Average length of isotigs | 535 bp | 513 bp |
| Range of isotigs length | 101-4,941 bp | 101-5,032 bp |
| Depth on isotigs | 2.1 | 1.03 |
|  |  |  |
| Singletons | 376,482 | 245,536 |
| Unique sequence | 409,789 | 274,987 |
