# Supplementary material for: Transcriptome sequencing and annotation of the microalgae Dunaliella tertiolecta: Pathway description and gene discovery for production of next-generation biofuels
Source: BMC Genomics. 2011 Mar 14;12:148. doi: 10.1186/1471-2164-12-148 (PMC3061936; doi:10.1186/1471-2164-12-148)
Supplement: Additional file 2 — A phylogenetic tree inferring the evolutionary relationship between D. tertiolecta and model microalgae Volvox carteri, and Chlamydomonas reinhardtii. The tree was generated using 18 S rRNA gene sequences of D. tertiolecta, Volvox carteri, and Chlamydomonas reinhardtii extracted from the NCBI database. Sequences were aligned using ClustalX and bootstrapping was performed in ClustalX with 100 iterations and values were displayed on the branch edges. The tree was visualized and published in Mega5. The distance bar represents 0.01 base changes/base. The tree was rooted with Methanobacterium congolense (NCBI Accession Number: AF233586.1). [file 1471-2164-12-148-S2.PDF]

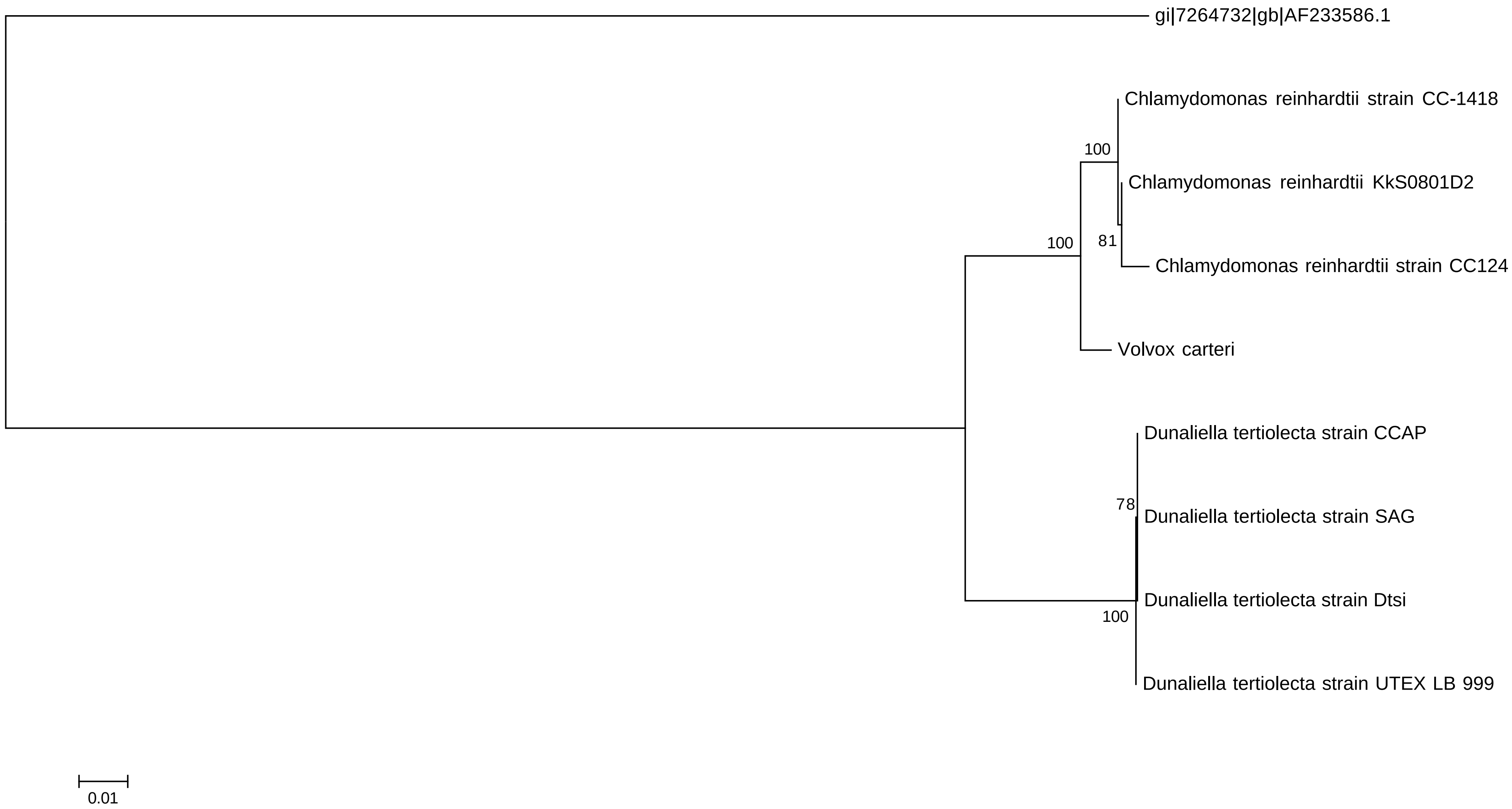
gi|7264732|gb|AF233586.1
Chlamydomonas reinhardtii strain CC-1418
100
Chlamydomonas reinhardtii KkS0801D2
100
81
Chlamydomonas reinhardtii strain CC124
Volvox carteri
Dunaliella tertiolecta strain CCAP
78
Dunaliella tertiolecta strain SAG
Dunaliella tertiolecta strain Dtsi
100
Dunaliella tertiolecta strain UTEX LB 999
0.01
