# Supplementary material for: Transcriptome sequencing and annotation of the microalgae Dunaliella tertiolecta: Pathway description and gene discovery for production of next-generation biofuels
Source: BMC Genomics. 2011 Mar 14;12:148. doi: 10.1186/1471-2164-12-148 (PMC3061936; doi:10.1186/1471-2164-12-148)
Supplement: Additional file 3 — Candidate genes identified based on KEGG orthology (KO) annotation of the D. tertiolecta transcriptome. [file 1471-2164-12-148-S3.DOC]

**Additional file 3:**  **Candidate genes identified based on KEGG orthology (KO) annotation of the *D. tertiolecta* transcriptome**

| KEGG-BRITE hierarchical classifications | KO Term | Number of sequences | Example (sequence ID) | UniProt ID (accession) |
| --- | --- | --- | --- | --- |
| **Genetic Information Processing** | | | | |
| RNA polymerase | 3020 | 58 | DNA-directed RNA polymerase II subunit A (isotig24153) | A8IRZ2 |
| Basal transcription factors | 3022 | 7 | Transcription initiation factor TFIIB (GHEXJPO01B87OM) | Q00VJ4 |
| Aminoacyl-tRNA biosynthesis | 00970 | 79 | Lysyl-tRNA synthetase, class I (isotig29735) | A8IPE8 |
| Protein export | 03060 | 44 | Translocation protein subunit SEC63 (isotig22815) | A8J8J1 |
| DNA replication | 03030 | 45 | DNA polymerase III beta-subunit (isotig27354) | A4AT48 |
| **Environmental Information Processing** | | | | |
| ABC transporters | 02010 | 60 | Nitrate transport protein (isotig14542) | Q0VR99 |
| Phosphatidylinositol signaling system | 04070 | 15 | Phosphatidylinositol 3-kinase (isotig27970) | O04269 |
| **Cellular Processes** | | | | |
| Peroxisome | 04146 | 38 | Peroxin-19 (isotig18280) | A8JGS1 |
| Regulation of autophagy | 04140 | 11 | unc51-like kinase (isotig24994) | A8J1A7 |
| Cell cycle | 04110 | 26 | Minichromosome maintenance protein 6 (isotig20217) | Q3E8H3 |
