# Supplementary figures and images for: Transcriptome sequencing and annotation of the microalgae Dunaliella tertiolecta: Pathway description and gene discovery for production of next-generation biofuels
Source: BMC Genomics. 2011 Mar 14;12:148. doi: 10.1186/1471-2164-12-148 (PMC3061936; doi:10.1186/1471-2164-12-148)

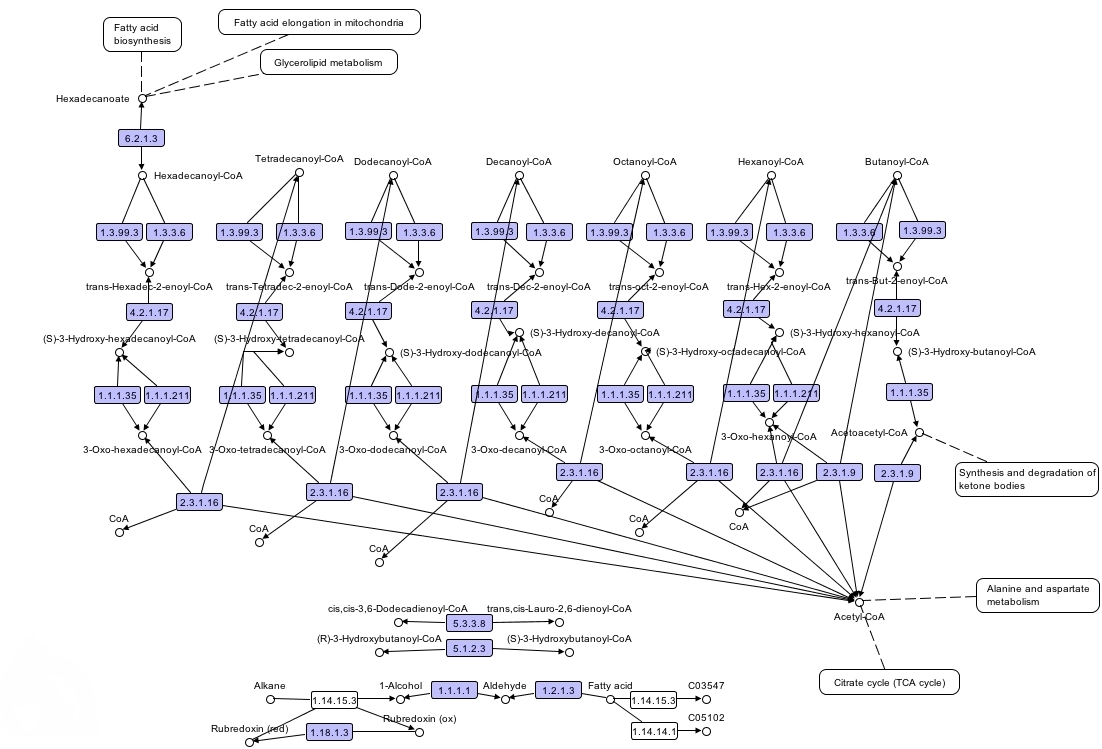

Supplement: Additional file 4 — β-oxidation pathway of D. tertiolecta based on the annotation of transcriptome and KEGG pathway assignment. [file 1471-2164-12-148-S4.JPEG]

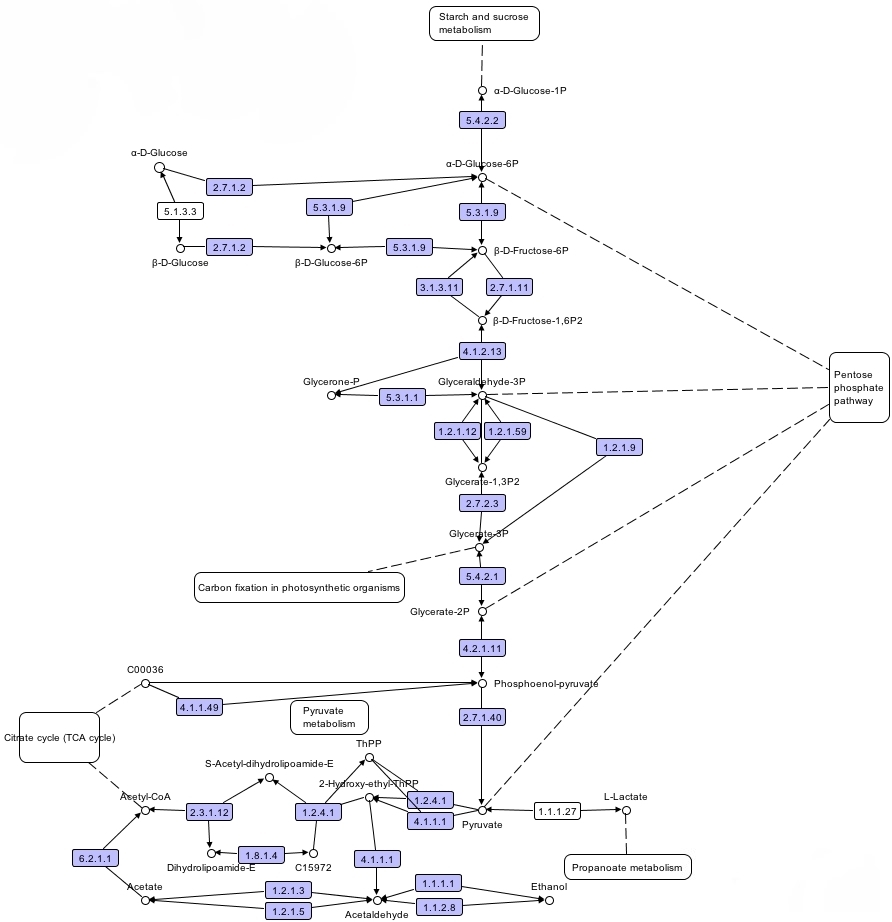

Supplement: Additional file 5 — Glycolysis pathway of D. tertiolecta based on the annotation of transcriptome and KEGG pathway assignment. [file 1471-2164-12-148-S5.JPEG]
