# Supplementary material for: Transcriptome sequencing and annotation of the microalgae Dunaliella tertiolecta: Pathway description and gene discovery for production of next-generation biofuels
Source: BMC Genomics. 2011 Mar 14;12:148. doi: 10.1186/1471-2164-12-148 (PMC3061936; doi:10.1186/1471-2164-12-148)
Supplement: Additional file 6 — Enzymes involved in glycolysis identified by annotation of the D. tertiolecta transcriptome. [file 1471-2164-12-148-S6.DOC]

**Additional file 6:** **Enzymes involved in glycolysis identified by annotation of the *D. tertiolecta* transcriptome**

| Enzyme | Symbol | EC Number | Number of transcripts |
| --- | --- | --- | --- |
| Phosphoglucomutase | PGM | 5.4.2.2 | 10 |
| Glucokinase | GCK | 2.7.1.2 | 6 |
| Glucose-6-phosphate isomerase | G6PI | 5.3.1.9 | 15 |
| 6-Phosphofructokinase | 6PFK | 2.7.1.11 | 19 |
| Fructose-bisphosphatase | FBP | 3.1.3.11 | 29 |
| Fructose-bisphosphate aldolase | FBPAL | 4.1.2.13 | 12 |
| Triose-phosphate isomerase | TPI | 5.3.1.1 | 4 |
| Glyceraldehyde-3-phosphate dehydrogenase (phosphorylating) | GAPDH | 1.2.1.12 | 18 |
| Glyceraldehyde-3-phosphate dehydrogenase (NADP^+^) | GAPDH | 1.2.1.9 | 5 |
| Phosphoglycerate kinase | PGK | 2.7.2.3 | 9 |
| Phosphoglycerate mutase | PGAM | 5.4.2.1 | 2 |
| Phosphopyruvate hydratase | PPH | 4.2.1.11 | 9 |
| Phosphoenolpyruvate carboxykinase (ATP) | PEPCK | 4.1.1.49 | 13 |
| Pyruvate kinase | PK | 2.7.1.40 | 39 |
| Pyruvate decarboxylase | PDC | 4.1.1.1 | 1 |
| Pyruvate dehydrogenase (acetyl-transferring)^1^ | PDHB | 1.2.4.1 | 14 |
| Dihydrolipoyllysine-residue acetyltransferase^1^ | DLAT | 2.3.1.12 | 6 |
| Dihydrolipoyl dehydrogenase^1^ | DDH | 1.8.1.4 | 10 |
| Acetate-CoA ligase | AceCL | 6.2.1.1 | 4 |
| Aldehyde dehydrogenase [NAD(P)^+^] | ALDH | 1.2.1.5 | 1 |
| Aldehyde dehydrogenase (NAD^+^) | ALDH | 1.2.1.3 | 3 |
| Alcohol dehydrogenase | ADH | 1.1.1.1 | 4 |

^1^Three enzymes of pyruvate dehydrogenase (acetyl-transferring) (EC: 1.2.4.1), Dihydrolipoyllysine-residue acetyltransferase (EC: 2.3.1.12), and Dihydrolipoyl dehydrogenase (EC: 1.8.1.4), make up the pyruvate dehydrogenase complex (PDHC) that transforms pyruvate into acetyl-CoA through pyruvate decarboxylation.
